# Schistosomiasis and liver disease: Learning from the past to understand the present

**DOI:** 10.1002/ccr3.2922

**Published:** 2020-05-22

**Authors:** Eman Abdelghani, Rommel Zerpa, Gloria Iliescu, Carmen P. Escalante

**Affiliations:** ^1^ Division of Internal Medicine Department of General Internal Medicine The University of Texas MD Anderson Cancer Center Houston Texas USA

**Keywords:** gastroenterology, hepatology, infectious diseases, liver diseases, schistosoma, schistosomiasis

## Abstract

To diagnose schistosomiasis, past medical history review should include recent travel to or from an endemic area, a history of elevated liver enzymes as well as contact with contaminated sources of water or farm animals.

## Graphical abstract

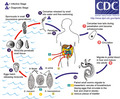

## 1 INTRODUCTION

Although schistosomiasis is common worldwide, it is rare in the United States. Lack of suspicion and poor screening methods can lead to severe complications. We present a case of liver schistosomiasis in a male Filipino patient living in the United States.

Schistosomiasis or “bilharziasis” named after its discoverer Theodor Bilharzis who first identified it in 1852, is a tropical disease caused by trematodes (flat‐worms) of the *Schistosoma* genus. Schistosomiasis is the second most common human parasitic infection causing approximately 280 thousand deaths annually due to complications such as renal failure and shock by hematemesis.^1^ It also causes a myriad of disabilities such as hematuria, kidney failure, bladder cancer, hydronephrosis, and portal hypertension.^2^ Schistosomiasis is second only to malaria in public health impact and in a global scale, it is estimated that 200 million people are infected by this parasite and more than 600 million are at risk.^3^ Currently, there are six identified *Schistosoma* species that can infect humans: *S japonicum*, *S mansoni, S haematobium, S intercalatum, S mekongi,* and *S malayensis*.^4^ Infection worldwide occurs mainly by the first three aforementioned species.

The fact that schistosomiasis has different reservoirs other than humans makes it difficult to eradicate in East and Southeast Asia, especially *S japonicum*.^5^ Although schistosomiasis is very common worldwide, it is very rare in the US but some cases arise from time to time, usually in immigrants from endemic areas. We present a case of liver schistosomiasis without developing major complications.

## 2 CASE HISTORY/EXAMINATION

A 47‐year‐old man who presented in November 2017 for abnormal liver enzymes and abnormal liver ultrasounds over the prior 2 years. He was noted to have abnormal liver function tests during his annual checkup in 2015. His anomalous results persisted, and he was diagnosed with fatty liver based on the laboratory results.

The patient had a past medical history that predisposed him to liver disease. He was born and raised in the Philippines on a farm where he was exposed to pesticides and pollution. He had cysticercosis during childhood, and received a Fuadin (Antimony) injection daily for 21 days with apparent resolution of disease. In 1997, while still living in the Philippines, it was suspected that he had tuberculosis, so he was placed on isoniazid and rifampin for 6 months. He also held a prior job that exposed him to bleach and formaldehyde. He last visited the Philippines 2 years earlier. He had no family history of liver disease. On physical examination, there were no abnormal findings. The liver edge was not palpable, and there was no tenderness.

## 3 INVESTIGATIONS

**FIGURE 1:**
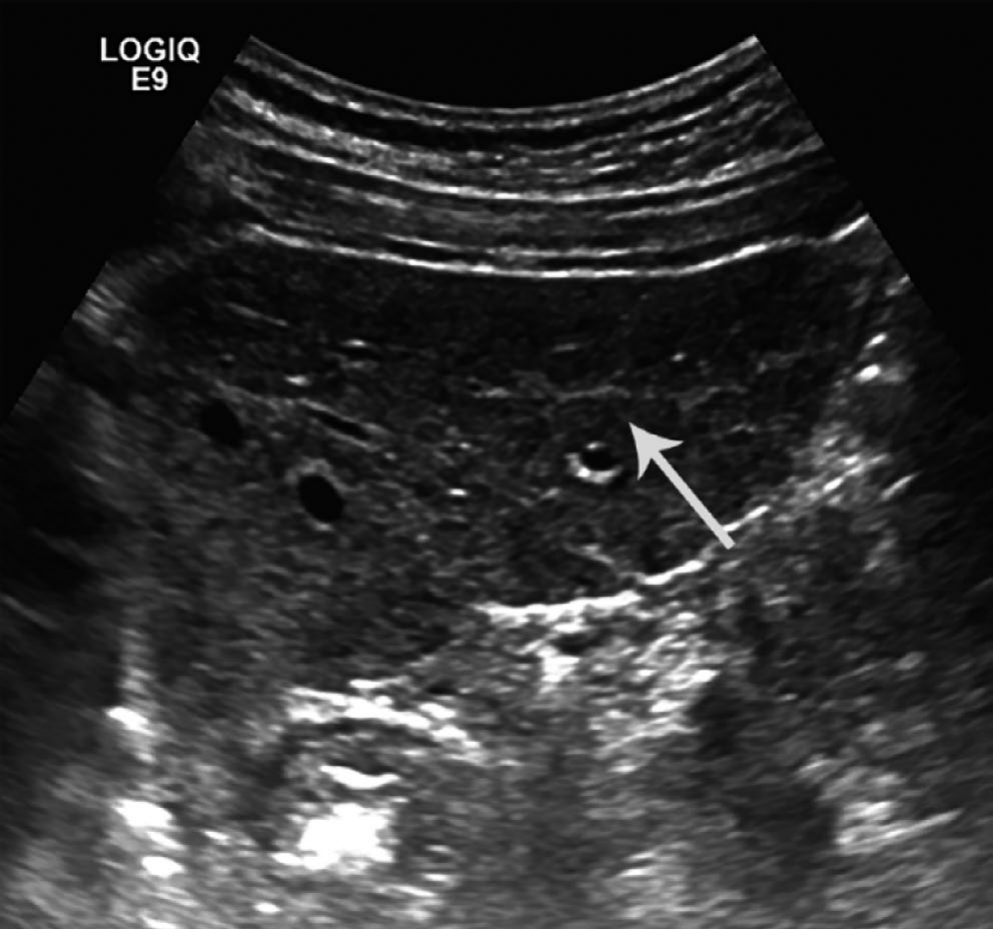
Hepatic fibrotic changes, Ultrasound

**FIGURE 2:**
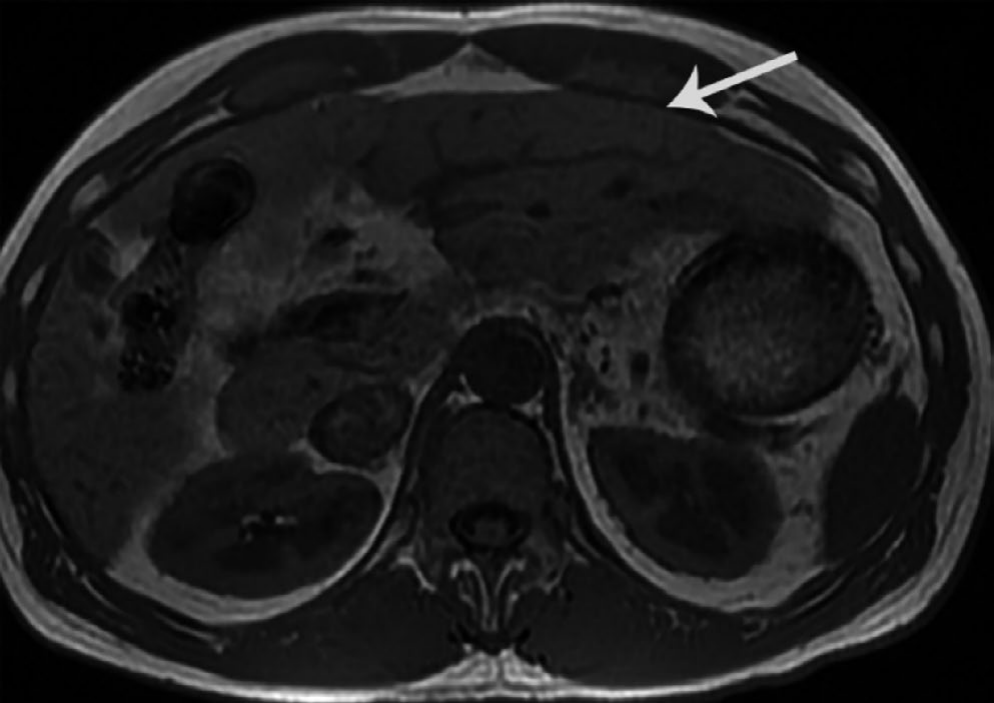
Hepatic surface nodularity, MRI

Laboratories revealed slightly elevated basophils (1.2% of a 7.7 WBC count), elevated AST (66 U/L, normal range 15‐46 U/L) and GGT (145 U/L, normal range 8‐78 U/L). Hepatitis viral panels were negative for hepatitis B and C. Iron studies, urine culture, complete metabolic and electrolytes were normal. A liver ultrasound was performed that showed an extremely heterogeneous parenchyma, which appeared to be interstitial fibrosis throughout the liver with increased septal lines throughout (Figure 1). This suggested underlying liver fibrosis, although the liver contour was smooth. Magnetic resonance imaging (MRI) of the abdomen with and without contrast was done, showing a slightly irregular outline of the liver with mild relative atrophy of the right lobe consistent with a background of chronic liver disease (Figure 2).

**FIGURE 3:**
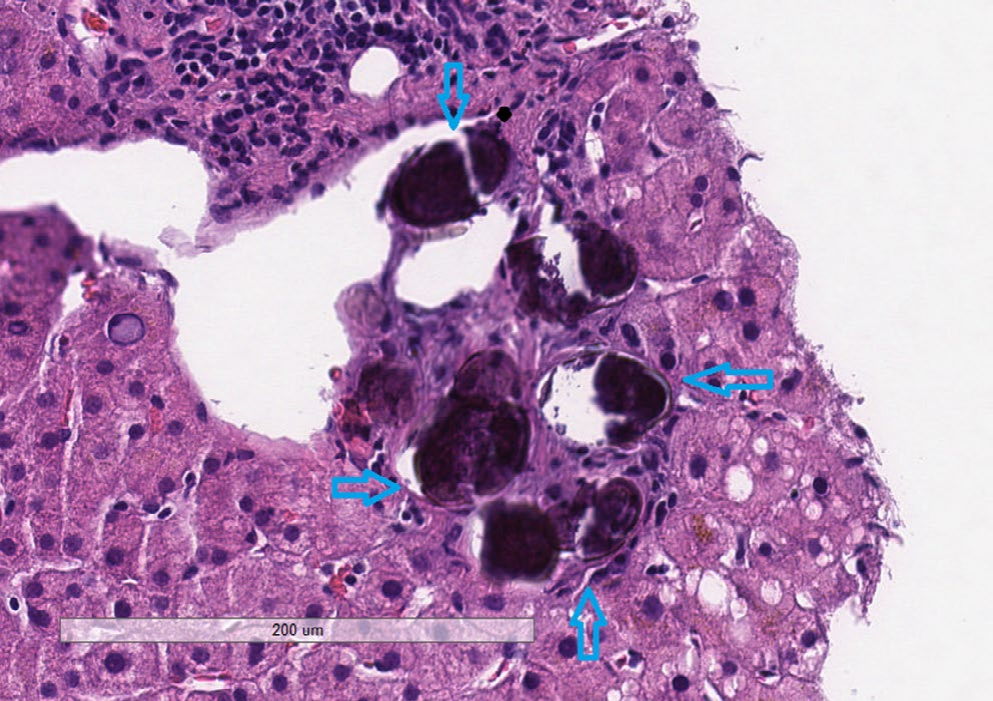
Calcified schistosoma ova, H&E stain

Since the patient had multiple risk factors that might contribute to his disease alongside remarkable imaging findings of liver fibrosis, gastroenterology was consulted and a liver biopsy was performed. The pathology report showed no evidence of cirrhosis but indicated the presence of a calcified schistosome ova in the portal tract with minimal inflammation and fibrosis (Figure 3), as well as minimal hepatocellular steatosis. A stool ova and parasite test was ordered and obtained on three separate days and were all negative for active infection.

## 4 DIFFERENTIAL DIAGNOSIS

The patient had a past medical history that predisposed him to liver disease. He was exposed to pesticides, pollution, had a history of cysticercosis during childhood, and presents with abnormal liver enzymes for 2 years. Given the lack of IV drug use, alcohol use disorder and unsafe sexual practices chronic hepatitis B or C, alcoholic hepatitis were less likely but nonalcoholic fatty liver disease was still considered in the picture. Biopsy findings ruled out the aforementioned diagnoses, enabling us to direct the treatment toward the parasitic etiology. (Table S1).^4^, ^5^, ^6^

## 5 TREATMENT

After confirming the diagnosis of liver schistosomiasis, treatment with praziquantel 600 mg oral three times a day for one day was recommended. An upper endoscopy and colonoscopy were also suggested to follow‐up and assess any complications arising from the disease alongside the digestive tract, as identifying them would be important in further treatment planning.

## 6 OUTCOME AND FOLLOW‐UP

A Liver ultrasound was performed 6 months later showing extremely heterogeneous liver parenchyma suggestive of chronic underlying hepatic pathology in the setting of known schistosomiasis and no specific sonographic evidence of portal hypertension.

Upper GI endoscopy was done on October 2018, showing normal esophagus, normal stomach, and normal duodenum, there was no esophageal or gastric varices seen and no evidence of portal gastropathy.

## 7 DISCUSSION

Schistosomiasis is a major public health problem, affecting more than 200 million people who acquire the infection through cercaria‐polluted water contact in 76 countries where the disease remains endemic. These countries are in South America, Africa and Southeast Asia.^5^ This disease was first reported in the Philippines in 1906. Currently, approximately, 865 000 people are infected with an additional 12 million at risk of infection. The greatest endemic foci are in the poorest regions of the Visayas and Mindanao. Some reports claim that the schistosomiasis control program of the Philippines is failing due to infrequent monitoring and evaluation, poor drug compliance, and coverage, and increasing reinfection rates.^7^, ^8^

Infection worldwide occurs mainly by the first three aforementioned species. Humans are the main reservoir and one of the definitive hosts, and contact with fresh water is the method of transmission. *S mansoni,* transmitted by *Biomphalaria* snails producing intestinal and hepatic schistosomiasis in Africa, the Arabian peninsula, and South America. *S haematobium*, has a reservoir in the *Bulinus* snails producing urinary schistosomiasis in Africa and the Arabian peninsula, and last, *S japonicum*, transmitted by the *Oncomelania* snail, producing intestinal and hepatosplenic schistosomiasis in China, the Philippines and Indonesia. Hepatic schistosomiasis can be caused *S mansoni* and *S japonicum* but the latter seems the more logical culprit in our case; this is a zoonotic parasite that infects cattle and other farm animals very often.^4^

**FIGURE 4:**
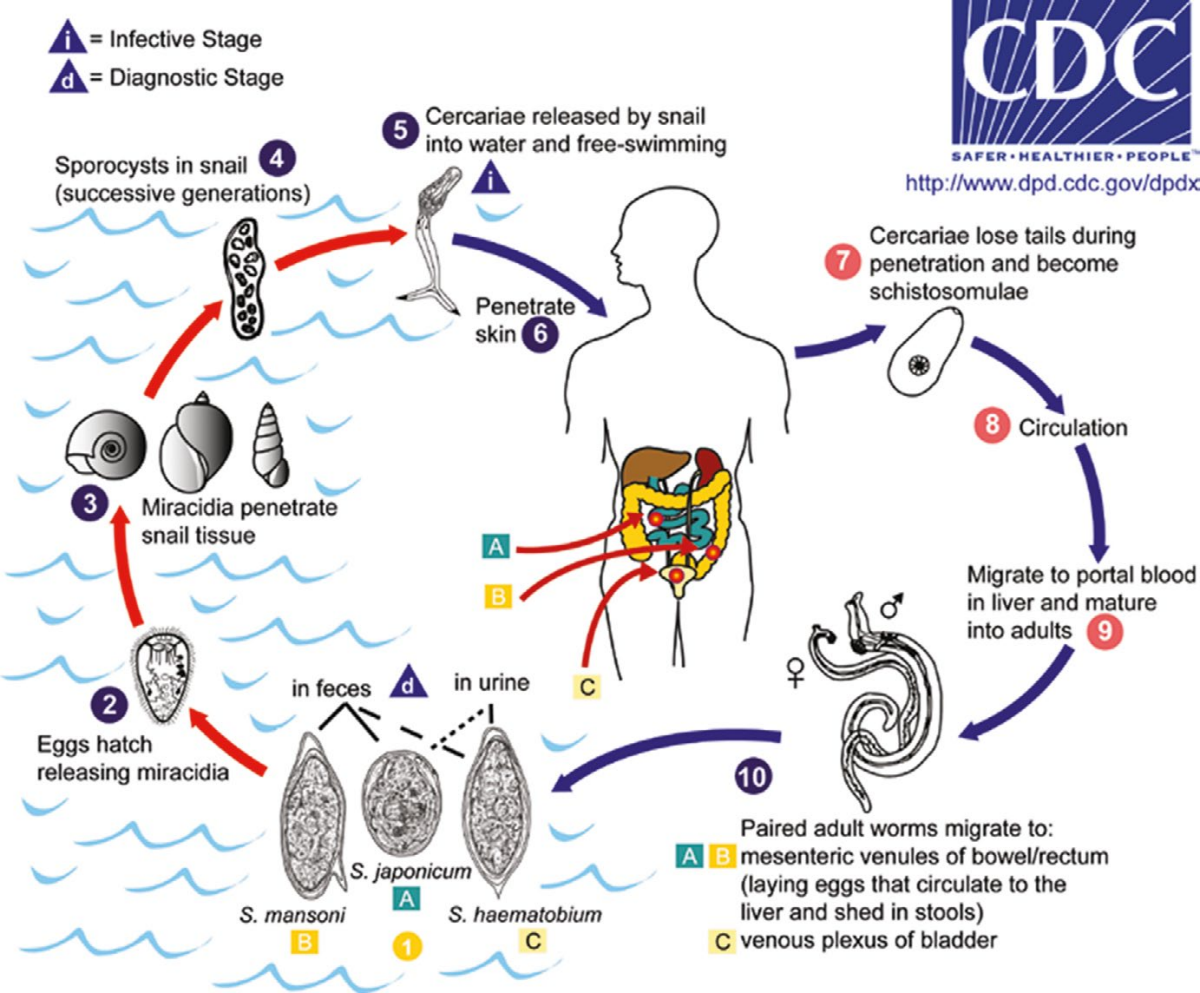
Schistosomiasis Life Cycle. Source: CDC, 2012^8^

The life cycle (Figure 4)^9^ of the parasite begins with the excretion of eggs in the urine (*S haematobium*) or feces (*S mansoni* or *japonicum*) into water and following this, eggs change into a miracidium larva. A fresh water snail is the intermediate host for the miracidium larva, where it transforms into sporocysts, then cercaria. The latter penetrates the skin of the humans in contaminated water and migrates to the liver through the hepatoportal circulation where it completes its maturation in the portal vein over 4‐6 weeks and then mates. Following, they travel to the perivesicular or mesenteric areas through blood.^4^

In this case, the patient grew up on a farm and had cysticercosis during childhood. He was treated with antimony injections and successfully cured, which raises suspicion as to whether he was also infected with schistosomes during childhood. During adulthood, he had mildly altered but persistent liver enzymes, with an ultrasound and MRI revealing fibrosis, and a final biopsy revealing a schistosome ova with surrounding fibrosis of the hepatic parenchyma. In this case, we are not certain how long the ova was in his liver given the lack of specificity for the time he was infected. It is stated that schistosomes can live up to 30 years in their definitive hosts, with a pair having a reproduction potential of, theoretically, around 600 billion schistosomes.^4^

All of the above findings suggest chronic infection. In chronicity, the lesions are not infringed by adult worms but instead by eggs that become trapped in the tissues during the perivesical or peri‐intestinal migration, as well as eggs that may embolize into the liver, lungs, spleen, or cerebrospinal system.^4^ Although the patient did not present with symptoms at the time of the visit, the minimally altered liver enzymes (AST 66 U/L and GGT 145 U/L) suggest an underlying hepatic process. There is always the possibility of developing cirrhosis with symptoms such as jaundice, fatigue, ascites, esophageal, or gastric varices, encephalopathy, increased bleeding, but this was not the case in our patient. His biopsy did not reveal cirrhosis. Upper endoscopy found no alterations at the level of the esophagus and his imaging findings matched those of limited parenchymal fibrosis of the liver. Therefore, it is important to consider schistosomiasis in a differential diagnosis in a patient with alteration of liver enzymes (Table S2),^10^ especially in those whose origin lie in endemic countries or have a history of traveling. Emphasis in screening this infection should be made given that treatment with antiparasitic drugs at therapeutic doses does not alter the progression of fibrosis.

Of note, some studies consider certain diets as altering the progression of fibrosis in schistosome‐infected patients. Sharaf EL‐Deen et al (2017), researched the antiparasitic and hepatoprotective properties of artichoke leaf extract (ALE) on mice experimentally infected with *S mansoni* and compared its effectiveness to praziquantel (PZQ). He concluded that despite failure of ALE to treat *S mansoni* infection it could limit liver damage by said parasite by modulating hepatic stellate cell recruitment.^11^

Screening should be used toward the prevention of progressive hepatic fibrosis in infected individuals. Ultrasound remains a low‐cost, convenient and reliable method, and is routinely used in the diagnosis and evaluation of patients with liver schistosomiasis, demonstrating classical features of schistosomal hepatic damage such as, shrinkage, perivascular thickening and fibrosis with characteristic “bullseye” lesions, a network echogenic pattern, granulomas and thickening of the gallbladder.^12^, ^13^ CT and MRI are methods absent in poorer socio‐economic environments, but the availability of ultrasound portable equipment has increased the applicability of imaging in community field settings in areas where schistosomiasis is endemic.^13^

## CONFLICT OF INTEREST

None declared.

## AUTHOR CONTRIBUTION

Eman H Abdelghani: MD, First author, wrote the case report. Rommel G Zerpa: MD, Second author, assisted with writing and editing. Gloria D Iliescu: MD, Third author, assisted with editing. Carmen P Escalante: MD, Senior author.

## Supporting information

Table S1‐S2
