# Supplementary material for: Schistosomiasis and liver disease: Learning from the past to understand the present
Source: Clin Case Rep. 2020 May 22;8(8):1522–6. doi: 10.1002/ccr3.2922 (PMC7455423; doi:10.1002/ccr3.2922)
Supplement: Supplementary file 1 — Table S1‐S2 [file CCR3-8-1522-s001.docx]

**SUPPLEMENT**

| **Point of comparison** | ***S. mansoni*** | ***S. haematobium*** | ***S. japonicum*** |
| --- | --- | --- | --- |
| **Global Distribution[6]** | Sub-Saharan Africa, Middle East, S. America, Caribbean | North & Sub-Saharan Africa, Middle East, Turkey, India | Asia |
| **Disease produced [4]** | intestinal and hepatic schistosomiasis | Urinary schistosomiasis | Intestinal and hepatosplenic schistosomiasis |
| **Transmitted by[4]** | *Biomphalaria* snails | *Bulinus* snails | Amphibian snail Oncomelania |
| **Eradication[5]** | Large, anthroponotic and, therefore eradicable | Large, anthroponotic and, therefore eradicable | The *S. japonicum* species-complex infects additional mammalian species, ruminants and rodents form important natural reservoirs and present an obstacle to Schistosomiasis eradication in East and Southeast Asia. |

**Table S1 - Comparison between Schistosoma species**

| **Causes of liver fibrosis** | **Differential diagnosis** |
| --- | --- |
| Alcoholic liver disease | Aspartate transaminase ≥ 2 times alanine transaminase level in 70% of patients, |
| Viral hepatitis C | Anti–hepatitis C virus antibody  If positive, order hepatitis C virus RNA |
| Viral hepatitis B | Hepatitis B surface antigen  Hepatitis B core antibody  If either is positive, order hepatitis B virus DNA |
| Nonalcoholic fatty liver disease | Ultrasonography may show fatty change  May need biopsy to diagnose nonalcoholic steatohepatitis |
| Hemochromatosis | Ferritin ≥ 250 to 300 ng per mL in men, ≥ 200 ng per mL in women |
| Primary bili­ary cholangitis (primary biliary cirrhosis) | Cholestasis (elevated alkaline phosphatase and glucose tolerance test)  Antimitochondrial antibody positive |
| Primary scleros­ing cholangitis | Perinuclear antineutrophil cytoplasmic antibodies positive in 70% of patients  Frequently positive antinuclear antibodies, antismooth muscle antibodies, other antibodies |
| Autoimmune hepatitis | Positive Antinuclear antibody and/or antismooth muscle antibody |
| Alpha1-antitrypsin deficiency | Alpha1-antitrypsin phenotype |
| Wilson disease | Low serum ceruloplasmin |

**Table S2 – Common Causes of liver fibrosis & it’s differential diagnosis [12]**
